# Supplementary material for: rGO-WO3 Heterostructure: Synthesis, Characterization and Utilization as an Efficient Adsorbent for the Removal of Fluoroquinolone Antibiotic Levofloxacin in an Aqueous Phase
Source: Molecules. 2022 Oct 17;27(20):6956. doi: 10.3390/molecules27206956 (PMC9610797; doi:10.3390/molecules27206956)
Supplement: Supplementary file 1 [file molecules-27-06956-s001.zip › molecules-1867383-supplementary.pdf]

SUPPORTING INFORMATION

rGO-WO_3 heterostructure: Synthesis, characterization and utilization as an efficient adsorbent for the removal of fluoroquinolone antibiotic levofloxacin in an aqueous phase

Manjot Kaur[a], Shafali Singh[b], Surinder Kumar Mehta[a], Sushil Kumar Kansal[b*]

[a]Department of Chemistry and Centre of Advanced Studies in Chemistry, Panjab University, Chandigarh 160014, India

[b]Dr. S. S. Bhatnagar University Institute of Chemical Engineering and Technology, Panjab University, Chandigarh 160014, India

***Corresponding author**

S. K. Kansal: sushilkk1@pu.ac.in

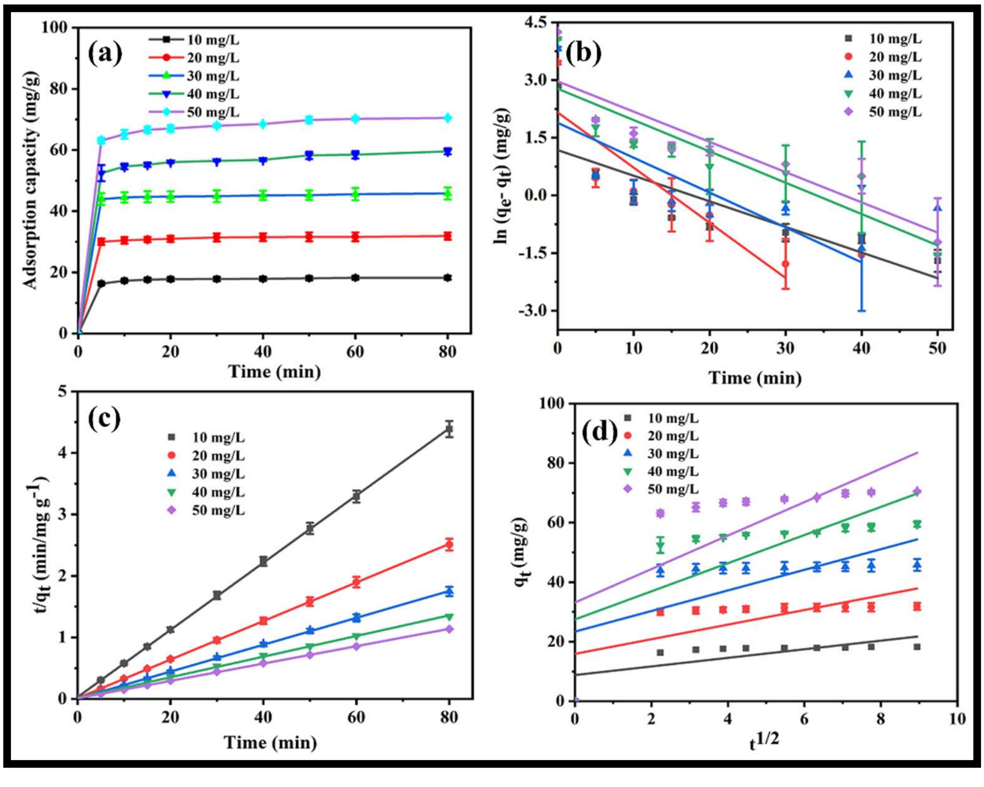

Figure S1. (a) Change in adsorption capacity of the prepared rGO-WO_3 heterostructure with respect to LVX concentration, (b) pseudo first order kinetic model, (c) pseudo second order kinetic model and (d) intraparticle diffusion model.

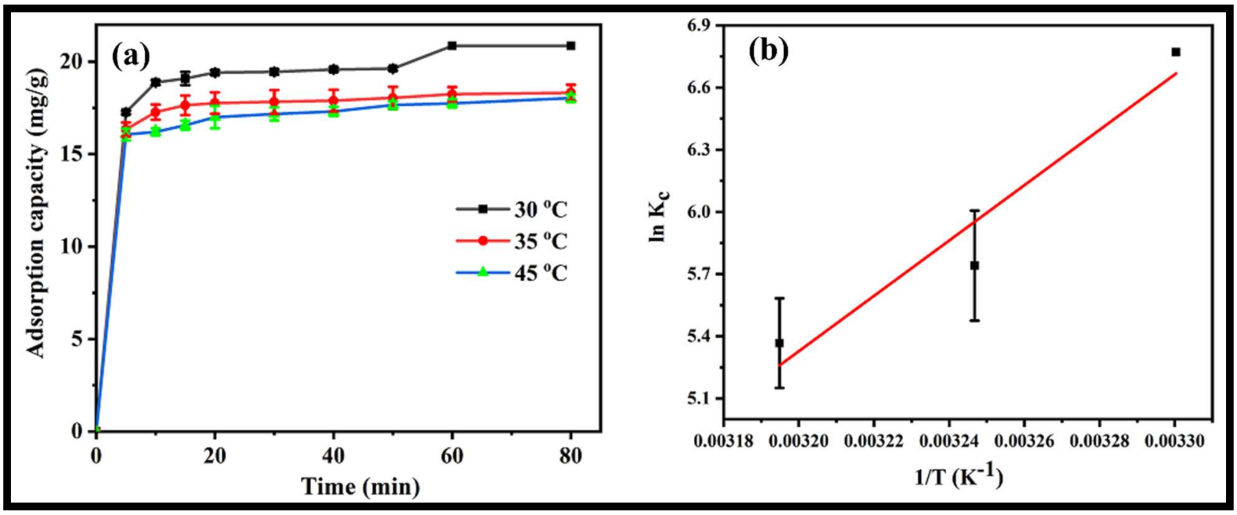

Figure S2. (a) Variation of adsorption capacity of the prepared rGO-WO_3 heterostructure with respect to temperature and (b) Van't Hoff plot for the adsorption of LVX over rGO-WO_3 heterostructure.

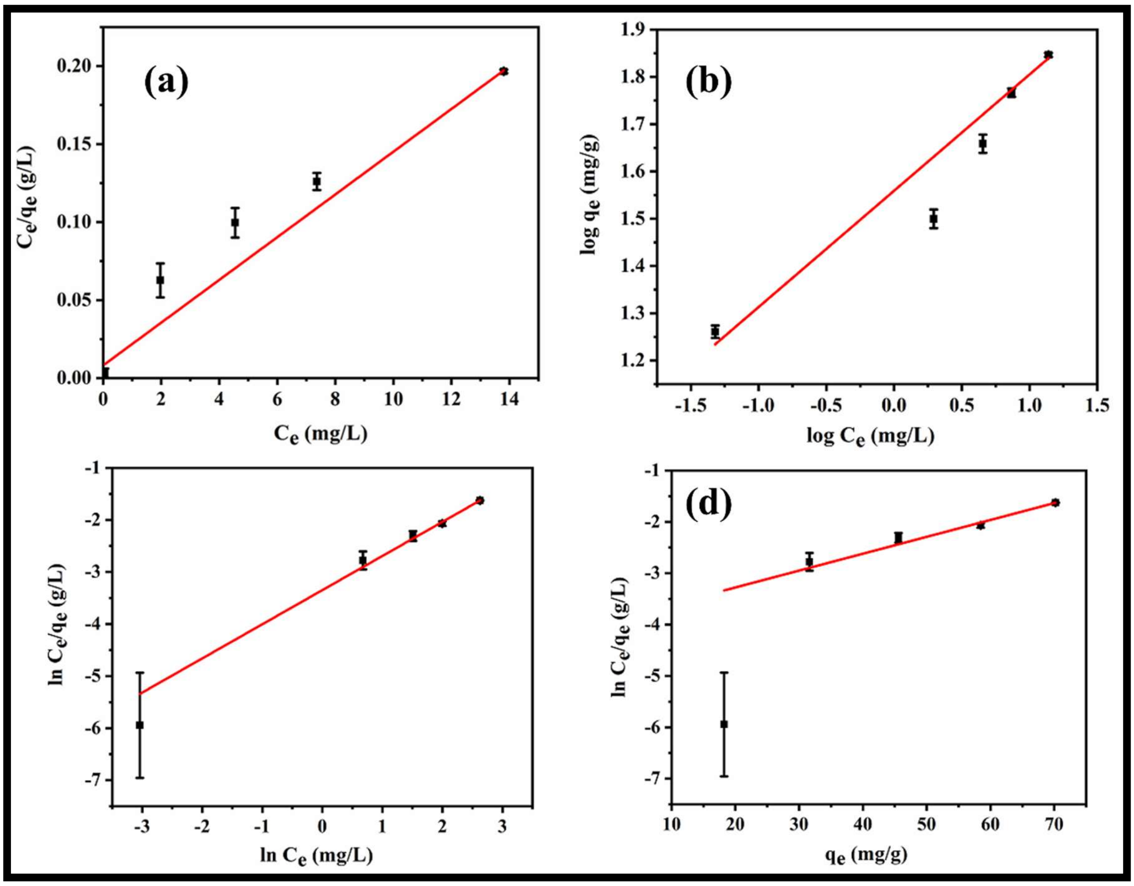

Figure S3. (a) Langmuir, (b) Freundlich, (c) Redlich-Peterson and (d) Jossen adsorption isotherms for LVX adsorption onto rGO-WO_3 heterostructure.

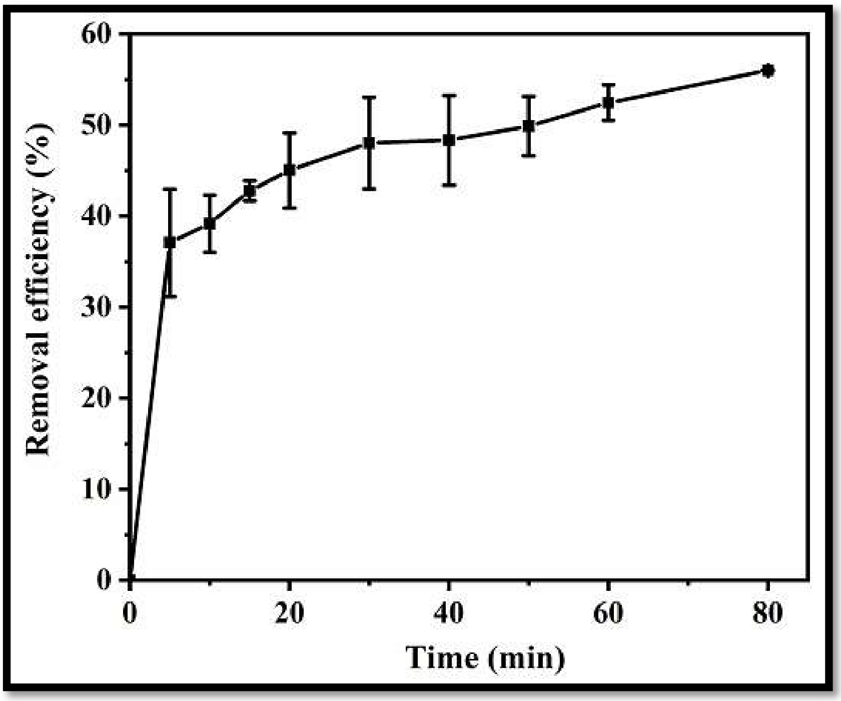

Figure S4. Adsorptive removal efficiency of rGO-WO_3 heterostructure in real water system spiked with LVX.

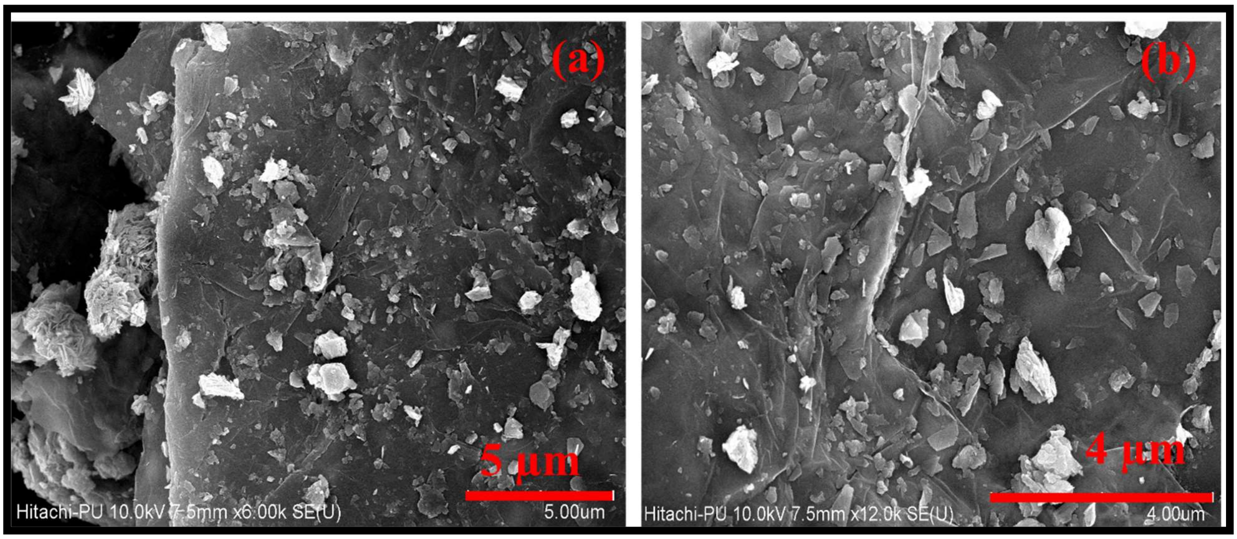

Figure S5. (a & b) FESEM images of the reused rGO-WO_3 heterostructure.
